# Sympathy

**Published:** 1887-01-15

**Authors:** 


### WORDS OF CONSOLATION.

[Communications for this column should be addressed, "Chaplain," care of the Editor of THE HOSPITAL, who will gratefully welcome any suggestions or inquiries from matrons, nurses, and the staff generally.]

### XVI.—SYMPATHY IN THE PHYSICIAN.

IT may be asked: What time have many medical men for shewing sympathy, even if they have the inclination? Suppose a physician has to visit several wards in a Hospital, or has to see such a quantity of out-patients in a given time that he knows he cannot do justice to their requirements, simply for want of time to gauge all their symptoms. Why, it is as much as he can do to prescribe hurriedly, or give the most essential directions, and pass on from case to case as rapidly as possible. What room has he for shewing much sympathy or personal interest in separate cases? No—this would be impossible were sympathy to be given as a prescription, or administered from time to time like a cordial. Where sympathy is real it has become part of a man's nature, and beams forth from him naturally and continually, without any studied effort to force it, either in manner or conversation. If it be our nature to sympathise, people will pass through our hands, and receive some of sympathy's sweet consolation, as certainly as they absorb sun-rays in the warmth of a summer's day. We might remind medical men, who feel they have more cases than they can do justice to, even physically, of the multitudes of sick folk pressing upon our Lord, so much so that at times He had no leisure to eat. Surely He had no time to treat every separate case with the attention they must have wished for. Numbers benefited must have longed for more personal converse with Him. Nevertheless we believe that many of the poor creatures, who had little direct intercourse with Him, drew consolation and hope from the very grace of His manner, and from the intense humanness which shone from His face. Thank God, we have perceived a measure of the same grace in some of our beloved physicians. The general tone and manner of the man are what tell so much upon hearts yearning for compassion.

How may those acquire more of this Divine gift who feel themselves wanting (and who does not?) "The way to get an increase of sympathy—says a well-known writer on sickness and its trials—"is to seek for increase of charity. The essence of sympathy is charity. No one without true charity can have godly sympathy. He who was perfect love had perfect sympathy. The more we are conformed to the image of perfect love, the more we really understand and seek to practise St. Paul's description of charity (1 Cor. xiii), the truer, and more abiding, and the deeper, will be our sympathy. If we are convinced that we do not understand the wants and trials of others, we shall ask Him to interpret them to us more fully."

Most medical men become aware, as they gain experience in their profession, that their popularity depends a good deal upon their manner in treating patients. But merely to study manners for the sake of popularity, is to risk their degenerating into "mannerism." Those who look upon their profession as a Divine calling will remember to pray daily for sympathy; and, having prayed, they will go forth on their mission of healing without any studied effort to maintain manners, but rather trusting in the Great Physician to manifest His sympathy through them as He wills, and when He wills.

(*To be continued.*)

